# Fabrication, Measurement and Time Decay of the Electromagnetic Properties of Semi-Solid Water-Based Phantoms

**DOI:** 10.3390/s19194298

**Published:** 2019-10-04

**Authors:** Carlos Mendes, Custódio Peixeiro

**Affiliations:** 1 Instituto Superior de Engenharia de Lisboa, Instituto Politécnico de Lisboa, 1959-007 Lisbon, Portugal; 2 Instituto de Telecomunicações, Instituto Superior Técnico, University of Lisbon, 1049-001 Lisbon, Portugal; custodio.peixeiro@lx.it.pt

**Keywords:** BAN, physical phantom, 2/3 muscle, coaxial probe technique, complex permittivity measurement

## Abstract

This paper presents a complete and detailed description of the fabrication and measurement of the electromagnetic properties of water-based semi-solid phantoms with emphasis on the analysis of the time evolution of the complex permittivity of several samples stored in different conditions. A known recipe for a 2/3 muscle equivalent phantom is used as test material, and the several phantom sample properties are measured with an in-house developed coaxial probe technique. It is shown that the storing condition is of paramount importance to extend the lifetime of a given phantom. This behavior stems from the way the storing condition affects the water evaporation rate of the sample. In particular, while an unprotected sample can preserve its electromagnetic properties only for a few days, a very well-sealed one can last at least up to a year.

## 1. Introduction

When the performance of an antenna placed near the human body must be measured, it is customary to use a phantom to mimic the electromagnetic properties of the human body. An excellent survey of different types of phantoms and how they can be fabricated can be found in [1]. For a phantom to be realistic it must mimic the electromagnetic properties of the human tissues in the frequency range of interest. As the main component of most biological tissues is water, the use of water as the main component of a phantom tends to guarantee a good approximation to the electromagnetic behavior of the envisaged biological tissue. By adding certain components to the water, according to an appropriated recipe, the water properties can then be modified to meet those of the intended tissue. One problem with a water-based phantom is its conservation as some evaporation will necessarily happen. To minimize the water loss and extend the lifetime span of the phantom it must be properly stored and handled. Therefore, the goal of this paper is to describe, in a complete and detailed way, the fabrication and measurement of the electromagnetic properties of water-based semi-solid phantoms and to access their lifetime span for different storing conditions.

The authors have recently presented a wearable fabric antenna [2,3] to work in the Industrial, Scientific and Medical (ISM) band at 2.45 GHz. The antenna was tested when placed on a homogeneous phantom mimicking a human torso with intended properties 2/3 of muscle, i.e., complex permittivity equal to 2/3 of that expected for muscle. At 2.45 GHz the muscle characteristics are $\varepsilon_{r}^{\prime }=52.73,\varepsilon_{r}^{\prime \prime }=12.77$ and tanδ=0.242 [4], where $\varepsilon_{r}^{\prime }$ and $\varepsilon_{r}^{\prime \prime }$ are, respectively, the real and imaginary parts of the relative complex permittivity and tanδ is the loss tangent. Therefore, the 2/3 muscle equivalent tissue is characterized by $\varepsilon_{r}^{\prime }=35.15,\varepsilon_{r}^{\prime \prime }=8.51$ and tanδ=0.242. This model has proven to be a good approximation for a layered inhomogeneous phantom with skin, fat, muscle and bone [5,6,7,8]. The material used in the fabrication of the torso phantom presented in [2,3] was also used in the samples analyzed in this work.

The organization of the paper is as follows: in Section 2 the recipe and fabrication procedure are described. Section 3 describes in detail the coaxial open-ended measurement technique used. The presentation of the experimental results obtained and an analysis of the time degradation of the dielectric properties are presented in Section 4. Final conclusions are given in Section 5. An uncertainty analyses is presented in the Appendix A.

## 2. Homogeneous Phantom Fabrication

The human body is made of many different tissues with unique electromagnetic properties. The characterization of these tissues has already been thoroughly studied by several researchers and their characterizations at different frequencies are currently available. A comprehensive study of body tissue characterization is presented in [9] where the complex permittivity of various human tissues is measured for frequencies ranging from 10 Hz to 100 GHz and fitted to a 4 term Cole–Cole model. When performing measurements, the intricate details of the human body are often replaced by a homogeneous volume representing a part of the human body and with a permittivity of some kind of average behavior of the real material in the intended frequency range.

In the literature there are several formulas [10,11,12] than can be used to produce phantoms mimicking the electromagnetic properties of biological tissues. The ingredients used and the fabrication procedure follow that described in [12]. This procedure was selected since it is based on cheap and easy to obtain ingredients, has a fabrication procedure which requires minimal equipment and allows easy adjustment of the electromagnetic properties of the phantom. Moreover, it leads to phantoms with mechanical (rheological) properties suitable for the envisaged applications [1,12]. Also, the phantom produced can take any desired shape since it will conform to the shape of the container where it will be stored. This was particularly important for the work presented by the authors, where the shape on a human torso was targeted to mimic the transmission between antennas placed on the torso.

Deionized water (the dominant ingredient), agar, polyethylene powder, sodium chloride (NaCl), TX-151 and sodium azide $\left({\mathrm{NaN}}_{3}\right)$ are the ingredients used which allow the obtaining of a final material that simulates the characteristics of the high-water-content human tissues, such as muscle, brain or internal organs, although not so suitable for low-water-content tissues such as fat and bone. In this phantom, retention of the shape is made possible with agar which also prevents water from separating. The polyethylene powder is used to adjust the relative permittivity while the conductivity is mainly adjusted by the concentration of sodium chloride. Since the agar solution and the polyethylene powder cannot be mixed directly, TX-151 is used to increase the viscosity. Sodium azide is added as a preservative. Using different proportions of the ingredients, this recipe has successfully been used to realize different materials. In [12], muscle and brain equivalent materials for frequencies up to 2.5 GHz are created. In [13], the same recipe is used to obtain a head-equivalent phantom for the 3–6 GHz frequency range, a real-shaped phantom of the upper half of the body at 2.6 GHz and an abdomen phantom of a pregnant women at 150 MHz. In [14,15] a recipe for a material capable of representing muscle for frequencies up to 12 GHz with 2/3 muscle properties is used to represent a real-shaped phantom of the upper half of the body of an adult male. In [16,17] a skin equivalent realistic hand for 60 GHz is presented. A model for propagation around a cylinder of 2/3 muscle is proposed and tested in [18].

The fabrication procedure, adapted from [12], is as follows:

- Deionized water is placed on a kettle and heated on a gas burner. During the heating process, sodium chloride and sodium azide are added to the water. To prevent evaporation, the heating process must be performed with the kettle’s lid on.
- Once the water starts to reach the boiling point, agar is slowly added and dissolved.
- When the mixture reaches the boiling point, fire is extinguished immediately.
- Small quantities of TX-151 and polyethylene powder are sprinkled into the liquid several times and quickly mixed with an electrical mixer at a speed fast enough to assure a uniform mixture but not so fast that air bubbles are formed.
- After all ingredients are added, the mixture is poured into the container, which must be immediately closed (with a plastic lid or cling-film) to prevent evaporation.
- Finally, the mixture is allowed to completely cool down to room temperature (for about a day) to completely solidify. After that period, the phantom can be removed from the container but must be kept in a hermetical environment to reduce water evaporation.

**Figure 1:**
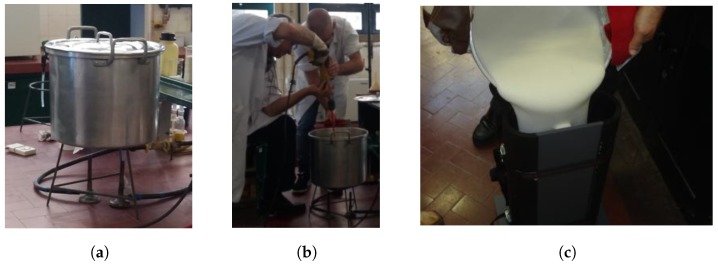
Photographs of the several steps of the phantom’s fabrication. (**a**) Water heating. (**b**) Ingredient mixing. (**c**) Container filling.

Photographs of the main steps of the phantom material fabrication are shown in Figure 1. These photographs detail the fabrication of the torso phantom used by the authors in [2,3], which is the same process used for the smaller samples.

**Figure 2:**
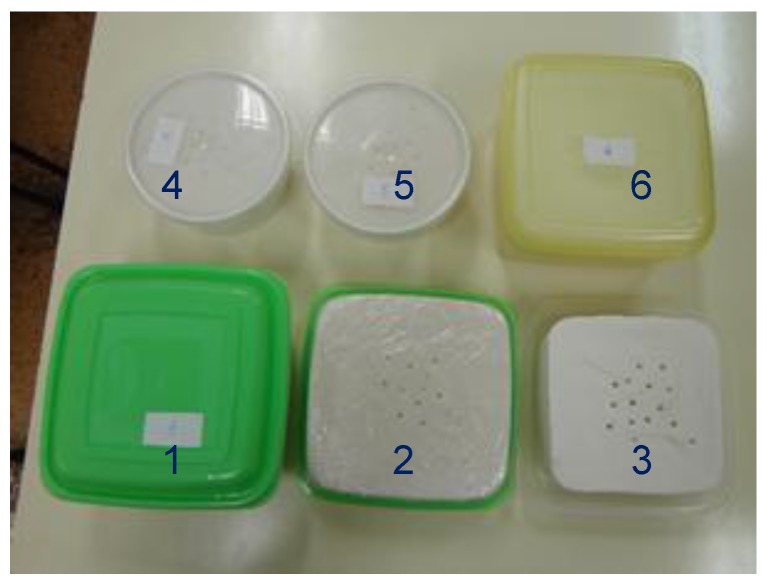
Photograph of the samples inside the containers.

Two batches of phantom material were fabricated, on two different occasions, with the quantities presented in Table 1. The smaller batch is directly obtained from [15] and aims at a material with 2/3 muscle properties in the range 2–9 GHz. This batch is used to produce several small test samples with different sizes and preservation conditions with the goal of measuring the degradation rate of the phantom’s properties with time for different storage conditions. The quantities of the larger batch are scaled from the smaller one to obtain a total of 12 L to build the torso phantom used in [3]. A small sample of this batch was also kept for measurements. The small samples and their respective containers/storing conditions are shown in Figure 2.

Sample 1 is approximately parallelepiped (average side 13.5 cm and average height 5.0 cm) and has been kept closed inside a plastic box. The air gap between the sample and the box cover is approximately 3.5 cm. Sample 2 and 3 are also approximately parallelepiped (average side 13.5 cm and average height 4.0 cm). Sample 2 has been kept wrapped in cling-film and sample 3 was left open without protection. Samples 4 and 5 are approximately cylindrical (average diameter 11 cm and average height 5.5 cm) and have been kept inside a plastic box with an air gap between sample and cover of 5 mm. Sample 6 is approximately parallelepiped with average side 13.5 cm and average height 6.5 cm and has been kept closed inside the plastic box for about one year. The air gap between the sample and the box cover is approximately 10 mm. The characteristics of the samples are summarized in Table 2.

## 3. Measurement Technique

The open-ended coaxial probe method [19,20,21,22,23] is used to measure the dielectric properties of the phantom. It is intended to get insight about the frequency ranges where the fabricated coaxial probes would provide useful results and where the fabricated phantom successfully mimics the human tissues. The equipment available at the time of the measurements limited the maximum usable frequency to 6 GHz.

### 3.1. Method Description

In simple media (linear, isotropic, homogeneous and time invariant) the complex permittivity is given by

(1) $$\varepsilon =\varepsilon_{0}\left[\varepsilon_{r}^{\prime }-j\left(\varepsilon_{r}^{\prime \prime }+\frac{\sigma_{dc}}{\omega \varepsilon_{0}}\right)\right]=\varepsilon_{0}\left(\varepsilon_{r}^{\prime }-j\varepsilon_{rgen}^{\prime \prime }\right)$$

where $\varepsilon_{0}$ is the vacuum permittivity, $\varepsilon_{r}^{\prime }$ is the real part of the relative complex permittivity, $\varepsilon_{r}^{\prime \prime }$ is the imaginary part of the relative complex permittivity, accounting for dielectric polarization losses, $\sigma_{dc}$ is the DC conductivity, ω is the angular frequency and $\varepsilon_{rgen}^{\prime \prime }$ is the total imaginary part of the relative complex permittivity.

**Figure 3:**
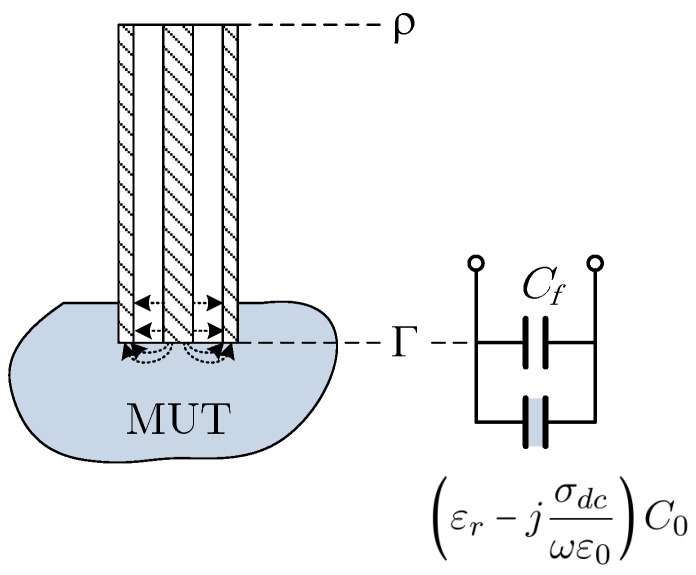
Equivalent circuit at the coaxial-MUT interface [21], where $\varepsilon_{r}=\varepsilon_{r}^{\prime }-j\varepsilon_{r}^{\prime \prime }$, $C_{f}$ is the fringing capacity at the tip of the probe, $C_{0}$ is the probe terminal capacity when in free space and ρ is the voltage reflection coefficient measured at the side of the probe connected to the vector network analyzer.

The theory behind the open-ended coaxial method for measuring the electrical properties of materials is very well established [19,20,21,22,23]. It consists of the use of an open-ended coaxial probe immersed in the material under test (MUT) and to measure the reflection coefficient in a reference plane. Figure 3 shows the coaxial open-ended probe immersed in the MUT and the equivalent circuit of the coaxial-MUT interface [21]. The reflection coefficient at the open-ended side of the coaxial probe is given by

(2) $$\Gamma =\frac{Z_{L}-Z_{0}}{Z_{L}+Z_{0}}=\frac{Y_{0}-Y_{L}}{Y_{0}+Y_{L}}$$

where $Z_{L}$ ($Y_{L}$) is the load impedance (admittance) at the probe tip and $Z_{0}$ ($Y_{0}$) is the coaxial probe characteristic impedance (admittance). The load admittance of the equivalent circuit is [19,21]

(3) $$\begin{array}{cc} Y_{L} & =j\omega C_{f}+j\omega \left(\varepsilon_{r}-j\frac{\sigma_{dc}}{\omega \varepsilon_{0}}\right)C_{0}\\ \; & =\omega C_{0}\left(\varepsilon_{r}^{\prime \prime }+\frac{\sigma_{dc}}{\omega \varepsilon_{0}}\right)+j\omega C_{0}\left(\varepsilon_{r}^{\prime }+\frac{C_{f}}{C_{0}}\right) \end{array}$$

resulting on

(4) $$\Gamma =\frac{1-\left(\varepsilon_{r}^{\prime \prime }+\frac{\sigma_{dc}}{\omega \varepsilon_{0}}\right)\omega Z_{0}C_{0}-j\omega C_{0}Z_{0}\left(\varepsilon_{r}^{\prime }+\frac{C_{f}}{C_{0}}\right)}{1+\left(\varepsilon_{r}^{\prime \prime }+\frac{\sigma_{dc}}{\omega \varepsilon_{0}}\right)\omega Z_{0}C_{0}+j\omega C_{0}Z_{0}\left(\varepsilon_{r}^{\prime }+\frac{C_{f}}{C_{0}}\right)}$$

**Figure 4:**
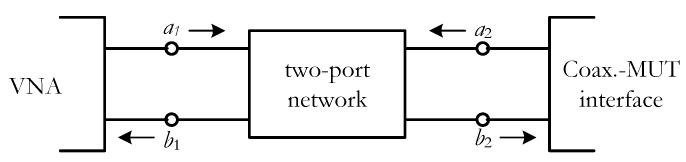
Two-port model of the coaxial probe [21].

The coaxial probe can also be modeled as a two-port network, as shown in Figure 4, and therefore it can be written

(5) $$\rho =\frac{b_{1}}{a_{1}}$$

(6) $$\Gamma =\frac{b_{2}}{a_{2}}=\frac{\rho -S_{11}}{S_{22}\left(\rho -S_{11}\right)+S_{12}S_{21}}$$

Equating (6) and (4) leads to

(7) $$\rho =\frac{A_{2}+A_{3}\left(\varepsilon_{r}^{\prime }-j\varepsilon_{rgen}^{\prime \prime }\right)}{A_{1}+\left(\varepsilon_{r}^{\prime }-j\varepsilon_{rgen}^{\prime \prime }\right)}$$

where

(8) $$A_{1}=\frac{1-S_{22}}{j\omega C_{0}Z_{0}\left(1+S_{22}\right)}+\frac{C_{f}}{C_{0}}$$

(9) $$A_{2}=\frac{S_{11}+S_{12}S_{21}-S_{11}S_{22}}{j\omega C_{0}Z_{0}\left(1+S_{22}\right)}+\frac{C_{f}}{C_{0}}\left(\frac{S_{11}-S_{12}S_{21}+S_{11}S_{22}}{1+S_{22}}\right)$$

(10) $$A_{3}=\frac{S_{11}-S_{12}S_{21}+S_{11}S_{22}}{1+S_{22}}$$

The complex coefficients $A_{1}$, $A_{2}$ and $A_{3}$ can be obtained from Equation (7) by terminating the coaxial probe with 3 standard media with known $\left(\varepsilon_{ri}^{\prime },\varepsilon_{rgeni}^{\prime \prime }\right)$ and measure the corresponding reflection coefficient $\rho_{i}$ (*i* = 1, 2, 3) at the VNA reference plane. The most common choice for the 3 standards is a short-circuit, an open-circuit and a well-known reference media. For the short-circuit (i=1), $\Gamma_{1}=-1$. Substituting in (6) gives

(11) $$-1=\frac{\rho_{1}-S_{11}}{S_{22}\left(\rho_{1}-S_{11}\right)+S_{12}S_{21}}$$

Solving for $\rho_{1}$ leads to

(12) $$\rho_{1}=\frac{S_{11}-S_{12}S_{21}+S_{11}S_{22}}{1+S_{22}}=A_{3}$$

For the open-circuit (i=2), $\varepsilon_{r2}^{\prime }=1,\varepsilon_{rgen2}^{\prime \prime }=0$. Substituting in (7) yields

(13) $$\rho_{2}=\frac{A_{2}+A_{3}}{A_{1}+1}$$

Finally, substituting again in (7) for a reference liquid with $\left(\varepsilon_{r3}^{,}\prime \varepsilon_{rgen3}^{\prime \prime }\right)$ results on

(14) $$\rho_{3}=\frac{A_{2}+A_{3}\left(\varepsilon_{r3}^{\prime }-j\varepsilon_{rgen3}^{\prime \prime }\right)}{A_{1}+\left(\varepsilon_{r3}^{\prime }-j\varepsilon_{rgen3}^{\prime \prime }\right)}$$

Rearranging for the $A_{i}$ it can finally be obtained

(15) $$A_{1}=\frac{\left(\rho_{2}-\rho_{1}\right)+\left(\rho_{1}-\rho_{3}\right)\left(\varepsilon_{r3}^{\prime }-j\varepsilon_{rgen3}^{\prime \prime }\right)}{\rho_{3}-\rho_{2}}$$

(16) $$A_{2}=\frac{\rho_{3}\left(\rho_{2}-\rho_{1}\right)+\rho_{2}\left(\rho_{1}-\rho_{3}\right)\left(\varepsilon_{r3}^{\prime }-j\varepsilon_{rgen3}^{\prime \prime }\right)}{\rho_{3}-\rho_{2}}$$

(17) $$A_{3}=\rho_{1}$$

Once the $A_{i}$ are obtained from measurements with the reference loads, the coaxial probe can be loaded with the MUT.

(18) $$\rho_{MUT}=\frac{A_{2}+A_{3}\varepsilon_{rMUT}}{A_{1}+\varepsilon_{rMUT}}$$

Solving for $\varepsilon_{rMUT}$

(19) $$\varepsilon_{rMUT}=\frac{A_{2}-A_{1}\rho_{MUT}}{\rho_{MUT}-A_{3}}$$

### 3.2. Coaxial Probe Description

**Figure 5:**
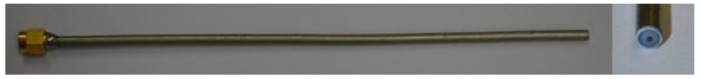
Photograph of the fabricated coaxial probe.

**Figure 6:**
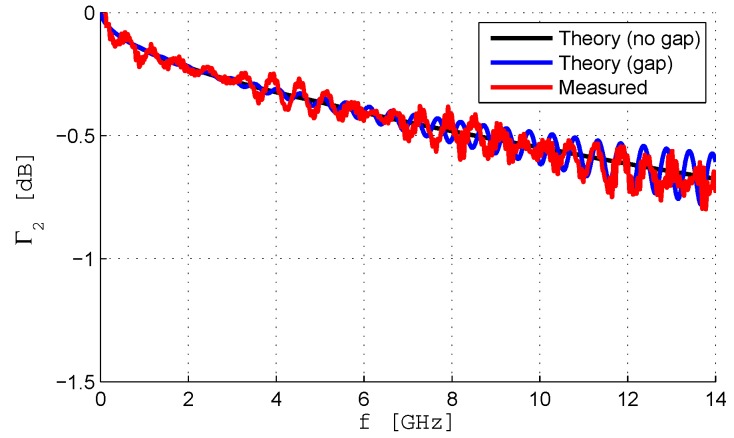
Input reflection coefficient of the coaxial probe.

A coaxial probe, shown in Figure 5, has been fabricated from the semi-rigid 50Ω coaxial cable UT141. The probe has a length of approximately 20 cm and is left open on one end and terminated with an SMA male connector on the other end to allow for the connection with a VNA. The measured input reflection coefficient of the open-ended probe is shown in Figure 6. An unexpected high ripple is noticed. The SMA connector can introduce small reflections and therefore some ripple was expected, but much smaller than the one obtained since the SMA connector is specified to be used up to 18 GHz. After a detailed analysis of the fabrication process it was concluded that a discontinuity had been introduced when soldering the cable section to the SMA connector. The high temperature needed to solder the cable to the connector led to the expansion of the dielectric insulator. After cooling, the dielectric retracted and an air gap was left between the dielectrics of the connector and of the cable. This air gap corresponds to a discontinuity in the cable characteristic impedance.

**Figure 7:**
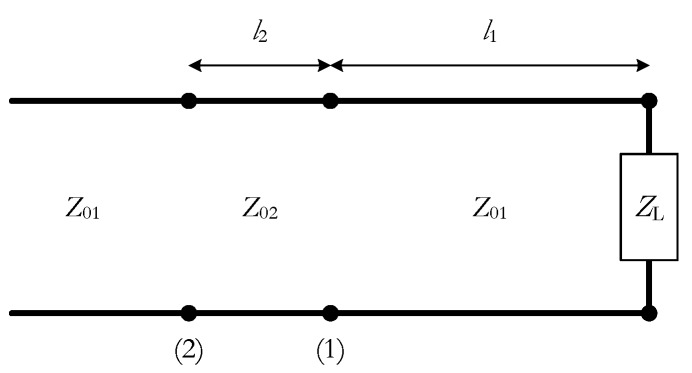
Transmission line model of the probe with air gap.

A simple transmission line circuit model can be used to check the effect of the air gap of the coaxial probe. The circuit model used is shown in Figure 7, where $l_{1}$ is the length of the probe and $l_{2}$ is the length of the air gap perturbation. As $l_{2}$ is very small (about 1 mm), and for the sake of simplicity, the corresponding section is considered lossless. Moreover, for the open-ended coaxial $Z_{L}=\infty$ will be assumed. Therefore, the impedance at the input of section 1 is given by

(20) $$Z_{in1}=Z_{01}\frac{e^{\gamma_{1}l_{1}}+e^{-\gamma_{1}l_{1}}}{e^{\gamma_{1}l_{1}}-e^{-\gamma_{1}l_{1}}}$$

with

(21) $$\gamma_{1}=\alpha_{1}+j\beta_{1}$$

The impedance at the input of the lossless section 2 can be obtained from

(22) $$Z_{in2}=Z_{02}\frac{Z_{in1}+jZ_{02}\tan\left(\beta_{2}l_{2}\right)}{Z_{02}+jZ_{in1}\tan\left(\beta_{2}l_{2}\right)}$$

Finally, the reflection coefficient at the input of section 2 is

(23) $$\Gamma_{2}=\frac{Z_{in2}-Z_{01}}{Z_{in2}+Z_{01}}$$

The amplitude of $\Gamma_{2}$ as a function of frequency is shown in Figure 6. The following data values have been used:

$$\begin{array}{ccc} Z_{01} & = & 50\;\Omega \\ l_{1} & = & 200\mathrm{mm}\\ \beta_{1} & = & \frac{2\pi }{\lambda_{1}}=\frac{2\pi f}{c}\sqrt{\varepsilon_{rPTFE}}\\ Z_{02} & = & Z_{0Air}=Z_{0PTFE}\sqrt{\varepsilon_{rPTFE}}=71.9\;\Omega \\ \beta_{2} & = & \frac{2\pi }{\lambda_{0}}=\frac{2\pi f}{c} \end{array}$$

The attenuation coefficient in section 1 of the cable has been obtained from typical UT141 data sheets and is reproduced in Table 3. The interpolation function indicated in Table 4 has been obtained from the data shown in Table 3.

The length of the air gap ($l_{2}=0.5$ mm) has been adjusted to reproduce the amplitude of the ripple obtained in the open-ended experimental response. The circuit model results are compared with measurements in Figure 6. Simulation results for the case without air gap ($l_{2}=0$) have also been introduced for reference. It seems that the attenuation is slightly underestimated above approximately 10 GHz. The adopted model of the air gap allows a proper reproduction of the high ripple obtained in the amplitude of the input reflection coefficient. The above-mentioned discrepancies are quite limited and, at least partially, have a physical explanation.

### 3.3. Coaxial Probe Calibration

For the probe calibration, besides the open-circuit and short-circuit, a third reference well-known media is needed. The use of at least a fourth reference well-known media is also advisable for the testing of the probe and calibration procedure. Although the authors worked with several well documented reference liquids (deionized water, methanol, ethanol, dimethyl sulfoxide (DMSO) and Water saline solutions) the results provided here were obtained with a calibration with deionized water. As methanol has permittivity values closed to the target phantom values, the uncertainty of methanol measurement will be calculated to obtain an estimate of the uncertainty of the measured phantom’s permittivity. An exchange of the role of these two liquids showed no relevant differences in the permittivity measurements.

**Figure 8:**
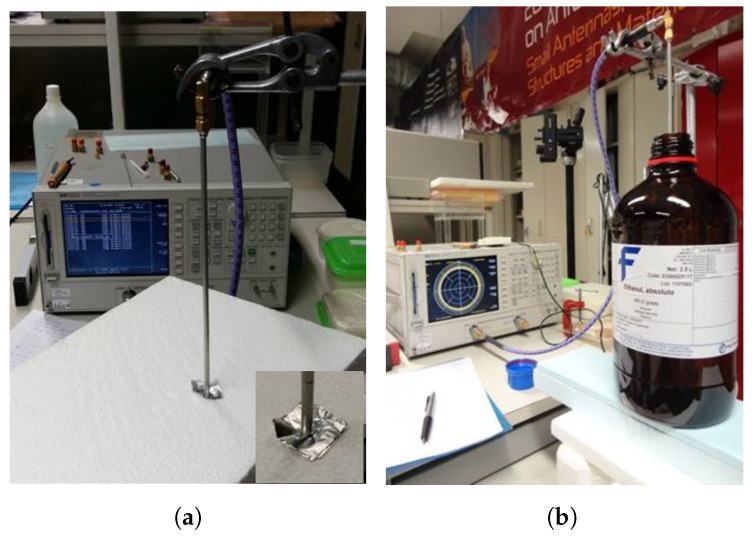
Experimental setup used for the measurements. The photographs show two examples, the short-circuit and a reference liquid. (**a**) Short-circuit. (**b**) Liquid.

The setup used in the measurements is shown in Figure 8a for the short-circuit and in Figure 8b for one of the reference liquids. A metallic holder was used to control the alignment and depth of the probe inside the liquids. During the measuring process it was noticed that the penetration depth of the probe was not a relevant parameter [24]. In fact, water measurements with a penetration of 20 mm, 40 mm and 60 mm are indistinguishable and only slightly different from the measurements with 0 mm. Methanol and ethanol provide experimental input reflection coefficient amplitude results that are also almost independent of the probe depth. However, a type of hysteresis effect was detected, i.e., for the same depth, the results obtained depended on whether the probe had just been inserted into the liquid or had stayed there for some time. As methanol and ethanol are less viscous than the other reference liquids used, and as the coaxial probe end is not sealed, some liquid could go inside the probe between the conductors (inner and outer) and the PTFE dielectric as was also identified in [20]. From the analysis of the results obtained it was decided to use a probe depth of 20 mm for all the reference liquids. Additionally, for methanol and ethanol measurements, special care was taken to make sure that the probe stays a short period of time inside the liquids to minimize infiltration into the probe.

**Figure 9:**
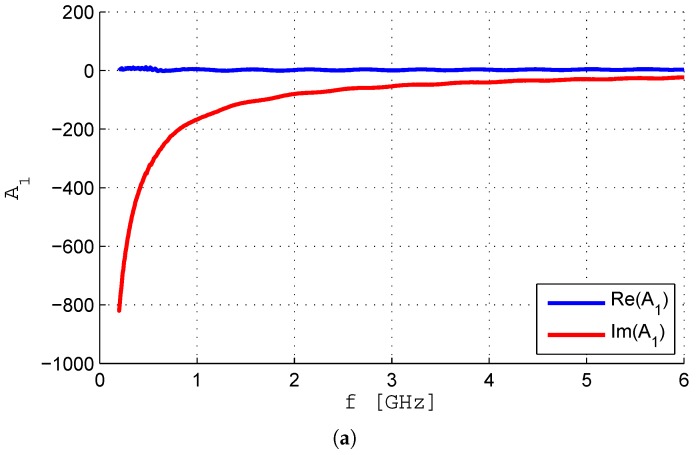

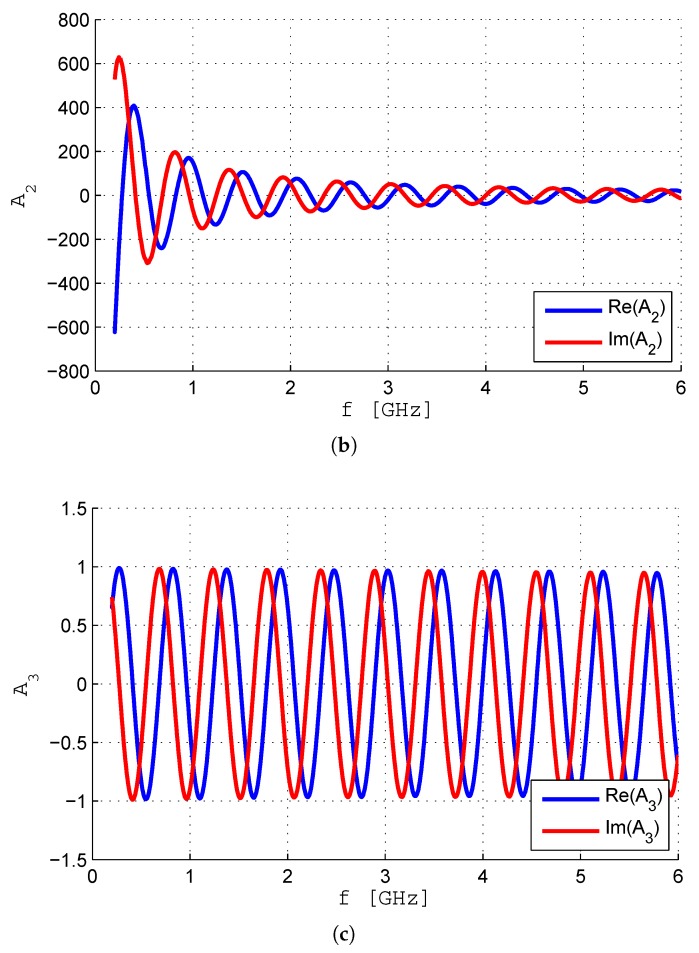
$A_{i}$ coefficients using deionized water as reference. (**a**) A1. (**b**) A2. (**c**) A3.

The coaxial open-ended coefficients $A_{1}$, $A_{2}$ and $A_{3}$, defined in Equations (8)–(10), respectively, have been obtained and are shown in Figure 9a–c. They have been measured in an environment with a stable temperature of 20.6 °C and 48% humidity.

## 4. Phantom Measurements

### 4.1. Comparison between Measured and Target Permittivity

The ultimate purpose is to measure the complex permittivity of the phantom material described previously. However, to test the repeatability of the fabrication process and also the way the permittivity changes with time with different storing conditions, six small samples of the phantom material, as shown in Figure 2, have also been fabricated. Samples 1 to 5 have been fabricated recently while sample 6 has been fabricated about one year before the current measurements. All the samples have been kept on a room with controlled temperature and humidity. In the first 18 days after fabrication the room temperature was 21±0.5 °C. On day 22 it was necessary to increase the temperature about 2 °C. The humidity of the room was in the range 50%±7%.

**Figure 10:**
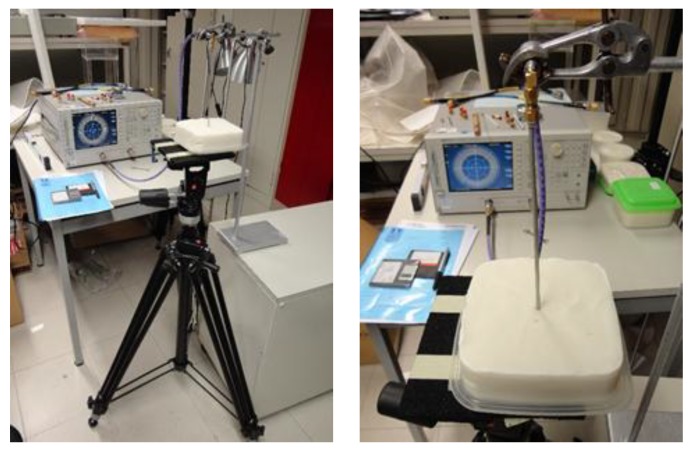
Photographs of the measurement setup used to measure the phantom samples using the sample left open as an example.

**Figure 11:**
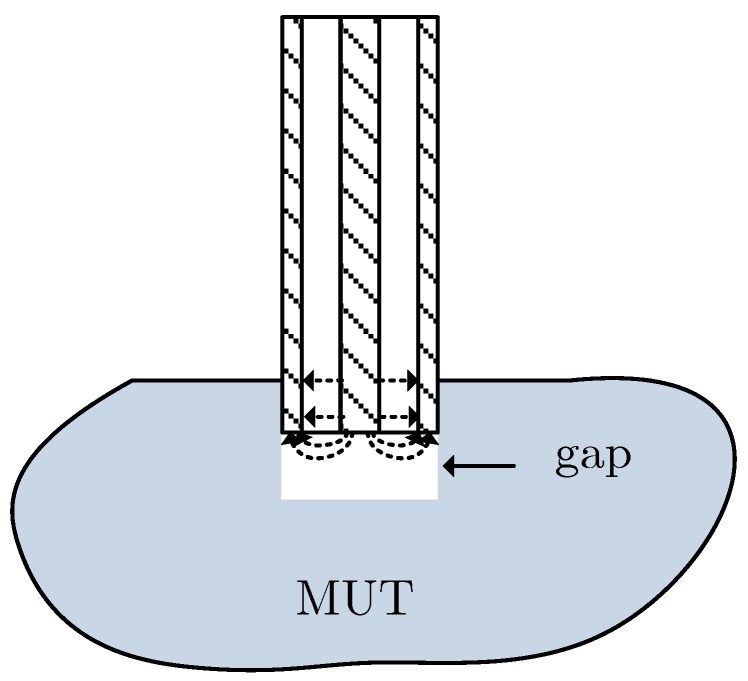
Accidental gap between the coaxial probe tip and the phantom material.

To measure the complex permittivity each sample was placed on the top of a photography tripod and moved up until the probe was about 9 mm deep inside the sample (Figure 10). It was necessary to move the sample smoothly, without going back and forth, to make sure that the sample material was in direct contact with the coaxial probe end. If there is a small gap between probe and sample material, as shown in Figure 11, either filled with air or water solution, the complex permittivity results will be substantially different [24].

**Figure 12:**
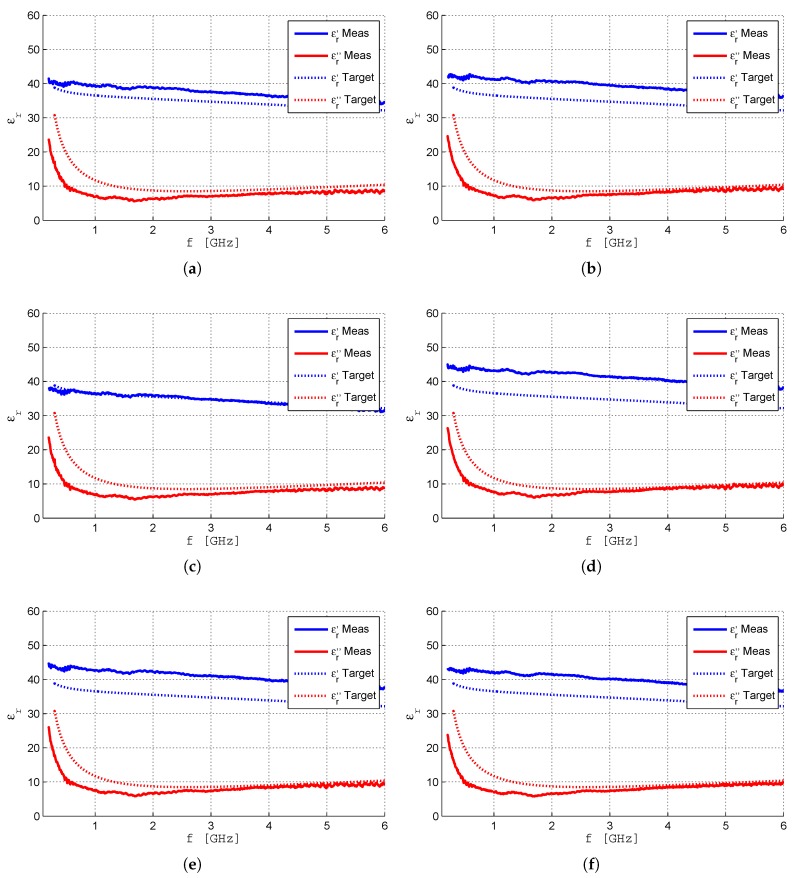
Measured and expected relative permittivity of the phantom samples. (**a**) Sample 1 (Parallelliped, Plastic). (**b**) Sample 2 (Parallelliped, Cling-film). (**c**) Sample 3 (Parallelliped, Open). (**d**) Sample 4 (Cilindrycal, Plastic). (**e**) Sample 5 (Cilindrycal, Plastic). (**f**) Sample 6 (Parallelliped, Plastic, one year).

The measured complex permittivity superimposed with the expected 2/3 muscle properties [4] are shown in Figure 12. All the results have been obtained 2 days after fabrication when the temperature of the samples was almost the same as the room temperature.

For the analysis of the results, it must be kept in mind that the recipe used in the phantom’s fabrication will produce a material with properties close to 2/3 muscle in the 2–9 GHz range. Therefore, the difference observed between the measured values and the target ones for frequencies below 2 GHz (specially noted in the values of $\varepsilon_{r}^{\prime \prime }$) is expected. There is a general very good agreement for the imaginary part of the relative complex permittivity of the six samples. The $\varepsilon_{r}^{\prime \prime }$ value is in the range 7.09–7.41 and tanδ is in the range 0.181–0.190. For the real part of the relative complex permittivity of the six samples the agreement is good between samples 2, 4, 5 and 6. Samples 1 and 3 have lower values of $\varepsilon_{r}^{\prime }$ meaning that they have lost water by evaporation. Sample 1, which is closed in a plastic box, lost some (little) water to the air volume (3.5 cm height) above the phantom material just until it got saturated. When the box is open, the water-saturated air is replaced by fresh air that, after the box is closed, becomes saturated again. This is the process by which the sample loses water. Sample 3, which is in a completely open situation, lost (more) water continuously. It is amazing that sample 6, despite being one year old, has preserved its macroscopic dielectric characteristics unchanged. This is because it was kept in a hermetically closed box that was never open in the one-year time period. The uncertainty analysis of the results obtained is described in Appendix A.

### 4.2. Time Evolution of the Phantom’s Characteristics

To evaluate the time evolution of the phantom dielectric characteristics, the several phantom samples were periodically measured. Regarding all the measurements, it is important to point out that the samples that are kept inside plastic boxes (1, 4, 5 and 6) are briefly open for the measurements and are closed hermetically afterwards and sample 2 is unwrapped for the measurements and is carefully wrapped immediately after.

**Figure 13:**
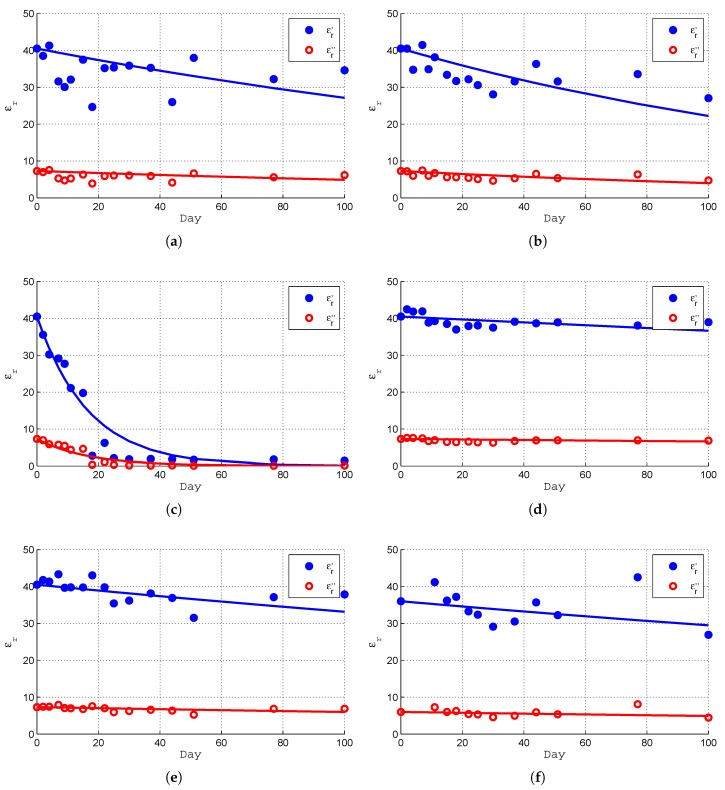
Time evolution of the phantom samples measured relative permittivity. (**a**) Sample 1 (Parallelliped, Plastic). (**b**) Sample 2 (Parallelliped, Cling-film). (**c**) Sample 3 (Parallelliped, Open). (**d**) Sample 4 (Cilindrycal, Plastic). (**e**) Sample 5 (Cilindrycal, Plastic). (**f**) Sample 6 (Parallelliped, Plastic, one year).

The time evolution of the experimental $\left(\varepsilon_{r}^{\prime },\varepsilon_{r}^{\prime \prime }\right)$ results are shown in Figure 13. The results shown indicate clear tendencies that depend on the type of protection of each of the phantom samples. As time goes by, if unprotected, the phantom samples rapidly lose water content and the relative permittivity goes down quickly. This effect can be observed in sample 3 where 11 days after fabrication $\varepsilon_{r}^{\prime }$ went down from about 40 to about 20 and $\varepsilon_{r}^{\prime \prime }$ from about 7 to about 4. However, there are some results that seem to be erroneous and do not follow any tendency. These results differ from the general tendency far above the uncertainty level of the measurement method. An example is, for instance, $\varepsilon_{r}^{\prime }$ of sample 1 on days 7, 9, 11, 18 and 44. Most of the “erroneous” results are below the tendency lines but there are also a few above. One possible explanation for the erroneous results is the fact that the coaxial probe tip might not be in direct contact with the sample material because of a small cylindrical gap, as depicted in Figure 11. This gap can be filled either with air, in which case the measured relative permittivity is lower than expected, or with water, in which case the measured relative permittivity is higher than expected. There are other factors contributing to the non-repeatability errors, such as the holes left by the previous measurements and the proximity of the measurement point to the phantom borders.

It should be pointed out that during the whole process, despite the water evaporation, the mechanical properties of the phantom are kept, i.e., the samples kept their shape and did not fall apart. The sample that was left open rapidly became noticeable dry. However, the remaining samples kept their original appearance during the whole process, which means that a visual inspection of the phantom is not enough to evaluate its current electromagnetic condition.

To be able to take some useful conclusions concerning the time evolution of the relative complex permittivity of the phantoms, the measured relative complex permittivity is fit to exponential tendency lines of the form

(24) $$\varepsilon_{r}\left(n\right)=\varepsilon_{r0}^{\prime }e^{-c_{1}n}-j\varepsilon_{r0}^{\prime \prime }e^{-c_{2}n}$$

where *n* is the number of days since the fabrication. The same values of $\varepsilon_{r0}^{\prime }$ and $\varepsilon_{r0}^{\prime \prime }$ are used in samples 1 to 5 and have been chosen as the average of the corresponding permittivity of samples 1, 2, 4 and 5 in day 2. Sample 6, being one year old, has been given a slightly different approach. It is quite natural that $\left(\varepsilon_{r}^{\prime },\varepsilon_{r}^{\prime \prime }\right)$ have both decreased a little during the one-year lifetime. In this case, $\varepsilon_{r0}^{\prime }=36$ and $\varepsilon_{r0}^{\prime \prime }=6$ have been used in the data fitting process. The values of $c_{i}$, obtained using a least-squares error minimization procedure, are indicated in Table 5.

The experimental results are compared with the fitted tendency lines of each sample in Figure 13. The following main conclusions can be drawn:

- Except for sample 3 (unprotected) the time decaying of the permittivity is almost linear.
- The real part $\varepsilon_{r}^{\prime }$ and the imaginary part $\varepsilon_{r}^{\prime \prime }$ of the permittivity have similar time decaying rates although a slightly faster decay of the imaginary part is noticed.
- The useful lifetime of an unprotected phantom is very small (a few days only).
- The most effective protection is a hermetic sealing box that can extend a phantom lifetime many months or even a year. This long extension of the lifetime is only possible if the volume of air is contact with the phantom is small and is not renewed.
- Wrapping a phantom in plastic film can also extend the useful lifetime of a phantom, not as much as a hermetic box, but it can reach up to a month.

Although these conclusions are obtained from the measurements of small samples of phantom material, we strongly believe that they can be qualitatively extrapolated to larger volumes such as the fabricated torso phantom.

## 5. Conclusions

A complete and detailed description of the fabrication and measurement of the electromagnetic properties of water-based semi-solid phantoms with emphasis on the analysis of the time evolution of the complex permittivity of several samples stored in different conditions is presented. It is shown that the storing conditions of a water-based phantom can largely influence the lifetime of the sample. If a sample is kept unprotected, its electromagnetic properties will last only for a few days. However, if hermetically stored, they will be preserved for at least a year. A sample that is kept hermetically closed and open regularly (in a daily basis) for a quick measurement can last up to a month. The phantom material is very lossy. Consequently, only the part near its surface has influence on the propagation over the phantom. Therefore, since the water evaporation rate is the main effect driving the time change of the permittivity and it is almost independent of the sample size, the conclusions obtained from the measurements of the small samples can be extrapolated to larger ones.

## Figures and Tables

**Table 1:** Composition of the 2/3 muscle equivalent material.

Ingredient	Amount (g)	Amount (g)
	(Original 4531.3 g)	(Scaled 12,000 g)
Deionized water	3375.0	8937.8
Agar	104.6	277.0
Polyethylene powder	1012.6	2681.6
Sodium chloride	7.0	18.5
TX-151	30.1	79.7
Sodium azide	2.0	5.3

**Table 2:** Summary of the characteristics of the several samples.

Sample	Shape	Storage	Age	Dimensions	Air Gap
				(cm)	(cm)
1	parallelepiped	plastic box	recent	13.5 × 13.5 × 5	3.5
2	parallelepiped	cling-film	recent	13.5 × 13.5 × 4	0
3	parallelepiped	open	recent	13.5 × 13.5 × 4	-
4	cylindrical	plastic box	recent	11 × 5.5	0.5
5	cylindrical	plastic box	recent	11 × 5.5	0.5
6	parallelepiped	plastic box	1 year	13.5 × 13.5 × 6	1.0

**Table 3:** UT141 typical attenuation.

Frequency (GHz)	Attenuation Coefficient (dB/m)
0.5	0.26
1	0.38
5	0.91
10	1.37
20	2.09

**Table 4:** UT141 cable interpolated attenuation.

Frequency (GHz)	Attenuation Coefficient (dB/m)
≤5	$0.047184\sqrt{f}-0.003434$
[1,5]	$0.049366\sqrt{f}-0.005616$
[5,10]	$0.057179\sqrt{f}-0.023086$
≥10	$0.063282\sqrt{f}-0.042385$

**Table 5:** Time constants of the permittivity tendency lines.

Sample	$c_{1}$	$c_{2}$
1	0.0039	0.0051
2	0.0050	0.0057
3	0.0700	0.0640
4	0.0010	0.0014
5	0.0016	0.0020
6	0.0036	0.0048

**Figure A1:**
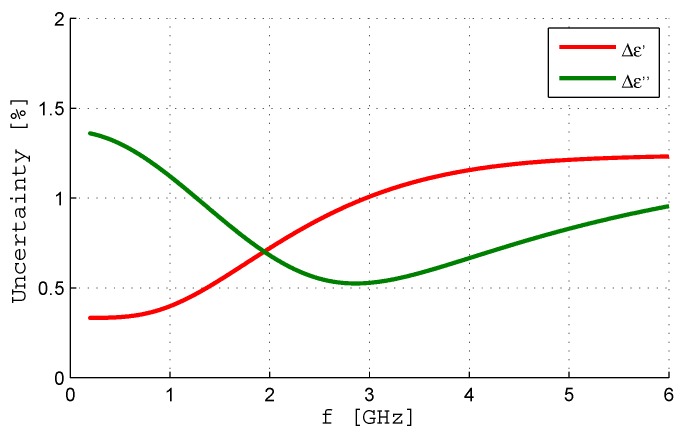
Uncertainty of methanol permittivity (T = 20.6 °C).

**Figure A2:**
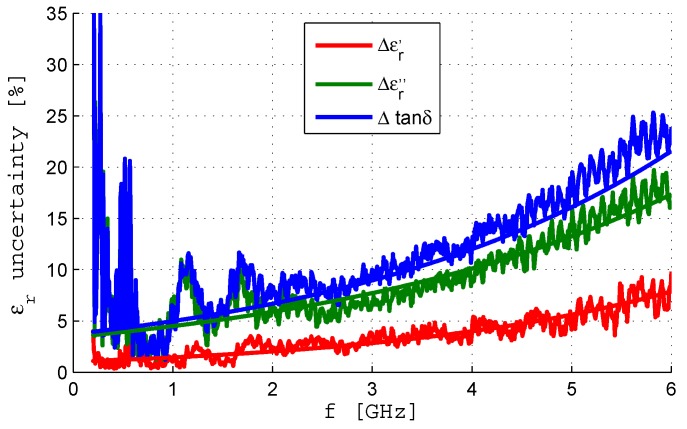
Uncertainty of methanol permittivity (T = 20.6 °C).

**Figure A3:**
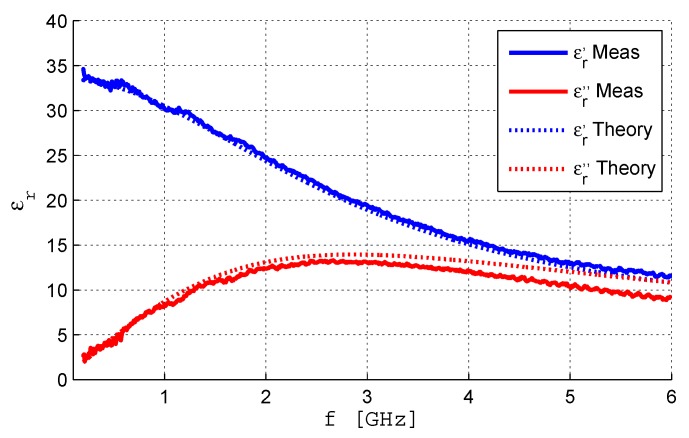
Theoretical and measured permittivity of methanol.

## Appendix A. Coaxial Probe Uncertainty Analysis

Depending on the frequency behavior of the permittivity, several models can be used to represent the liquids. A very common model is the Debye (or single-Debye) model [25,26,27], according to which the relative permittivity is given by

(A1) $$\varepsilon_{r}=\varepsilon_{\infty }+\frac{\varepsilon_{s}-\varepsilon_{\infty }}{1+j\omega \tau }=\varepsilon_{\infty }+\frac{\varepsilon_{s}-\varepsilon_{\infty }}{1+j\frac{f}{f_{r}}}$$

where $\varepsilon_{s}$ is the static (f=0) value of relative permittivity, $\varepsilon_{\infty }$ is the high frequency limit of the relative permittivity ($f>>f_{r}$), τ is the relaxation time, i.e., the time period needed for dipoles to revert to random orientation when the applied electric field is removed, ω is the angular frequency of the applied electric field, *f* is the linear frequency of the applied electric field and $f_{r}$ is the relaxation frequency $f_{r}=1/2\pi \tau$.

The relevant parameters needed to characterize methanol are contained in Table A1 [26] and they can be used for frequencies up to 10 GHz. For temperatures other than those in Table A1, all the Debye model parameters can be estimated from linear interpolation. For methanol, in the 20–25 °C range (where all the measurements were made) the interpolation process results on

(A2) $$\varepsilon_{s}=37.56-0.1967T$$

(A3) $$\varepsilon_{\infty }=6.018-0.0182T$$

(A4) $$f_{r}=1.546+0.0638T$$

**Table A1:** $\varepsilon_{s}$, $\varepsilon_{\infty }$ and τ values and uncertainty for methanol [26].

T (°C)	$\varepsilon_{s}\pm \Delta \varepsilon_{s}$	$\varepsilon_{\infty }\pm \Delta \varepsilon_{\infty }$	τ±Δτ (ps)
10	35.74±0.06	5.818±0.104	2.262±0.019
15	34.68±0.06	5.698±0.131	2.532±0.023
20	33.64±0.06	5.654±0.104	2.822±0.019
25	32.66±0.06	5.563±0.176	3.141±0.033
30	31.69±0.06	5.450±0.204	3.490±0.204

The theoretical Debye model has been obtained by curve fitting experimental data to the model and therefore have associated levels of uncertainty. Knowing the uncertainty of the several model parameters and using the concept of propagation of uncertainties [28] the uncertainty of the measured permittivity can them be estimated.

Separating the real and imaginary parts of the single-Debye model results on

(A5) $$\varepsilon_{r}^{\prime }=\varepsilon_{\infty }\frac{\varepsilon_{s}-\varepsilon_{\infty }}{1+{\left(f/f_{r}\right)}^{2}}$$

(A6) $$\varepsilon_{r}^{\prime \prime }=\frac{\left(\varepsilon_{s}-\varepsilon_{\infty }\right)\left(f/f_{r}\right)}{1+{\left(f/f_{r}\right)}^{2}}$$

The loss tangent is given by

(A7) $$\tan\delta =\frac{\varepsilon_{r}^{\prime \prime }}{\varepsilon_{r}^{\prime }}$$

The uncertainty of the previous quantities is related to the uncertainty on the model parameters by

(A8) $$\Delta \varepsilon_{r}^{{}^{\prime }}=\sqrt{{\left(\frac{\partial \varepsilon_{r}^{\prime }}{\partial \varepsilon_{\infty }}\Delta \varepsilon_{\infty }\right)}^{2}+{\left(\frac{\partial \varepsilon_{r}^{\prime }}{\partial \varepsilon_{s}}\Delta \varepsilon_{s}\right)}^{2}+{\left(\frac{\partial \varepsilon_{r}^{\prime }}{\partial f_{r}}\Delta f_{r}\right)}^{2}}$$

(A9) $$\Delta \varepsilon_{r}^{{}^{\prime \prime }}=\sqrt{{\left(\frac{\partial \varepsilon_{r}^{\prime \prime }}{\partial \varepsilon_{\infty }}\Delta \varepsilon_{\infty }\right)}^{2}+{\left(\frac{\partial \varepsilon_{r}^{\prime \prime }}{\partial \varepsilon_{s}}\Delta \varepsilon_{s}\right)}^{2}+{\left(\frac{\partial \varepsilon_{r}^{\prime \prime }}{\partial f_{r}}\Delta f_{r}\right)}^{2}}$$

(A10) $$\Delta \tan\delta =\sqrt{{\left(\frac{\partial \tan\delta }{\partial \varepsilon_{r}^{\prime }}\Delta \varepsilon_{r}^{\prime }\right)}^{2}+{\left(\frac{\partial \tan\delta }{\partial \varepsilon_{r}^{\prime \prime }}\Delta \varepsilon_{r}^{\prime \prime }\right)}^{2}}$$

The values of uncertainty of the model parameters are given for a discrete set of temperatures (for methanol they are given in Table A1). For temperatures in the range 20 °C to 25 °C the values of $\varepsilon_{s}$, $\varepsilon_{\infty }$ and $f_{r}$ are obtained from Equations (A2)–(A4), respectively. Therefore, $\Delta \varepsilon_{s}$, $\Delta \varepsilon_{\infty }$ and $\Delta f_{r}$ can be obtained from

(A11) $$\Delta \varepsilon_{\infty }=\sqrt{{\left(\frac{\partial \varepsilon_{\infty }}{\partial \varepsilon_{\infty }\left(25\right)}\Delta \varepsilon_{\infty }\left(25\right)\right)}^{2}+{\left(\frac{\partial \varepsilon_{\infty }}{\partial \varepsilon_{\infty }\left(20\right)}\Delta \varepsilon_{\infty }\left(20\right)\right)}^{2}+{\left(\frac{\partial \varepsilon_{\infty }}{\partial T}\Delta T\right)}^{2}}$$

(A12) $$\Delta \varepsilon_{\infty }=\sqrt{{\left(\frac{\partial \varepsilon_{s}}{\partial \varepsilon_{s}\left(25\right)}\Delta \varepsilon_{s}\left(25\right)\right)}^{2}+{\left(\frac{\partial \varepsilon_{s}}{\partial \varepsilon_{s}\left(20\right)}\Delta \varepsilon_{s}\left(20\right)\right)}^{2}+{\left(\frac{\partial \varepsilon_{s}}{\partial T}\Delta T\right)}^{2}}$$

(A13) $$\Delta f_{r}=\sqrt{{\left(\frac{\partial f_{r}}{\partial f_{r}\left(25\right)}\Delta f_{r}\left(25\right)\right)}^{2}+{\left(\frac{\partial f_{r}}{\partial f_{r}\left(20\right)}\Delta f_{r}\left(20\right)\right)}^{2}+{\left(\frac{\partial f_{r}}{\partial T}\Delta T\right)}^{2}}$$

Performing the partial derivatives using the values contained in Table A1 and assuming a temperature variation Δ*T* = 0.5 °C results on

(A14) $$\Delta \varepsilon_{\infty }=\sqrt{{\left[0.0352\left(T-20\right)\right]}^{2}+{\left[0.0208\left(25-T\right)\right]}^{2}+0.0091^{2}}$$

(A15) $$\Delta \varepsilon_{\infty }=\sqrt{{\left[0.012\left(T-20\right)\right]}^{2}+{\left[0.012\left(25-T\right)\right]}^{2}+0.098^{2}}$$

(A16) $$\Delta f_{r}=\sqrt{{\left[0.0066\left(T-20\right)\right]}^{2}+{\left[0.0038\left(25-T\right)\right]}^{2}+0.0319^{2}}$$

The theoretical uncertainty (in percentage) of the methanol single-Debye permittivity values, $\Delta \varepsilon_{r}^{\prime }$ and $\Delta \varepsilon_{r}^{\prime \prime }$, at 20.6 °C is shown in Figure A1 (these results agree with [28]).

An estimation of the uncertainty of the measured results can now be obtained as the difference between the measured result and the worst case of ($\varepsilon_{r}+\Delta \varepsilon_{r}$ or $\varepsilon_{r}-\Delta \varepsilon_{r}$). The results (in percentage) for $\Delta \varepsilon_{r}^{\prime }$, $\Delta \varepsilon_{r}^{\prime \prime }$ and Δtanδ are shown in Figure A2. The lower frequency intense ripple is caused by the air gap accidentally created between the PTFE insulation of the cable and the SMA connector (see Section 3.2). The higher frequency ripple is caused by other small multiple reflections (connector, cable connecting to the VNA, etc.). An exponential tendency line of the form

(A17) $$\Delta F\left(f\right)=Ae^{Bf}$$

has been added to the results were A and B, as indicated in Table A2, were obtained by a least square fit algorithm. As expected, the uncertainty increases as the frequency increases. At 2.45 GHz the uncertainties are approximately $\Delta \varepsilon_{r}^{\prime }=2.3\%$, $\Delta \varepsilon_{r}^{\prime \prime }=6.6\%$ and Δtanδ=7.6%

**Table A2:** Parameters of the uncertainty tendency lines.

Parameter	$\Delta \varepsilon_{r}^{\prime }$	$\Delta \varepsilon_{r}^{\prime \prime }$	Δtanδ
A	1.0250	3.4302	3.717
B	0.3398	0.2701	0.293

The measured permittivity superimposed with the expected values are shown in Figure A3. The measurements have been performed in stable conditions of temperature and humidity. As it can be seen there is a general good agreement between theoretical and experimental results with a tendency for the difference to increase as frequency increases. The relative differences in the permittivity at 2.45 GHz are $\Delta \varepsilon_{r}^{\prime }=0.491\left(2.3\%\right)$ and $\Delta \varepsilon_{r}^{\prime \prime }=0.536\left(3.9\%\right)$.
